# Silage Fermentation Quality, Anthocyanin Stability, and *in vitro* Rumen Fermentation Characteristic of Ferrous Sulfate Heptahydrate-Treated Black Cane (*Saccharum sinensis* R.)

**DOI:** 10.3389/fvets.2022.896270

**Published:** 2022-05-17

**Authors:** Ngo Thi Minh Suong, Siwaporn Paengkoum, Abdelfattah Zeidan Mohamed Salem, Pramote Paengkoum, Rayudika Aprilia Patindra Purba

**Affiliations:** ^1^School of Animal Technology and Innovation, Institute of Agricultural Technology, Suranaree University of Technology, Nakhon Ratchasima, Thailand; ^2^Department of Agriculture, School of Animal Sciences, Can Tho University, Can Tho, Vietnam; ^3^Program in Agriculture, Faculty of Science and Technology, Nakhon Ratchasima Rajabhat University, Nakhon Ratchasima, Thailand; ^4^Facultad de Medicina Veterinaria y Zootecnia, Universidad Autonoma del Estado de Mexico, Toluca, Mexico; ^5^Department of Health, Faculty of Vocational Studies, Airlangga University, Surabaya, Indonesia

**Keywords:** anthocyanin, cellulolytic bacteria, ferrous sulfate heptahydrate, gut microbiota, iron-treated silage, potential feedstuff, ruminant performance

## Abstract

Pretreatment of lignocellulose agricultural biomass with iron prior to ensiling is required to accelerate biomass breakdown during fermentation, which could result in functional microorganisms and chemicals that reduce nutrition loss, harmful substances, and improve animal performance. The objective of this study was to investigate the effects of increasing dilutions of ferrous sulfate heptahydrate (FS) pretreatment at fresh matter concentrations of 0, 0.015, and 0.030% on the fermentation quality of black cane (BC) silage, anthocyanin stability, ruminal biogas, rumen fermentation profile, and microbial community. Pre-ensiled and silage materials were evaluated. High moisture, fiber, anthocyanin, and lignification of biomass, as well as undesirable ensiling microorganisms, were found in BC' pre-ensiled form. Increasing dilutions of FS incorporated into silages were observed to linearly decrease dry matter, anthocyanin, and nutritive value losses. The lignin values decreased linearly as the percentage of FS increased up to 0.030%. Given that the ruminants were fed pre-ensiled materials, BC silage treated with 0.030% FS dilution had comparable results to pre-ensiled BC in terms of increasing *in vitro* volatile fatty acid concentrations, maintaining total gas production, and reducing methane production, when compared to other FS-treated silages. In addition, BC silage treated with a 0.030% FS dilution inhibited methanogenic bacteria and regulated cellulolytic bacteria in rumen fluid. Overall, the anthocyanin content of BC remained constant throughout the rumen fermentation process after increasing dilutions of FS, indicating that BC is a viable ruminant feedstock and that pretreatment of BC with dilute FS-assisted ensiling at 0.030% could be used to generate ruminant diets.

## Introduction

**Figure 1:**
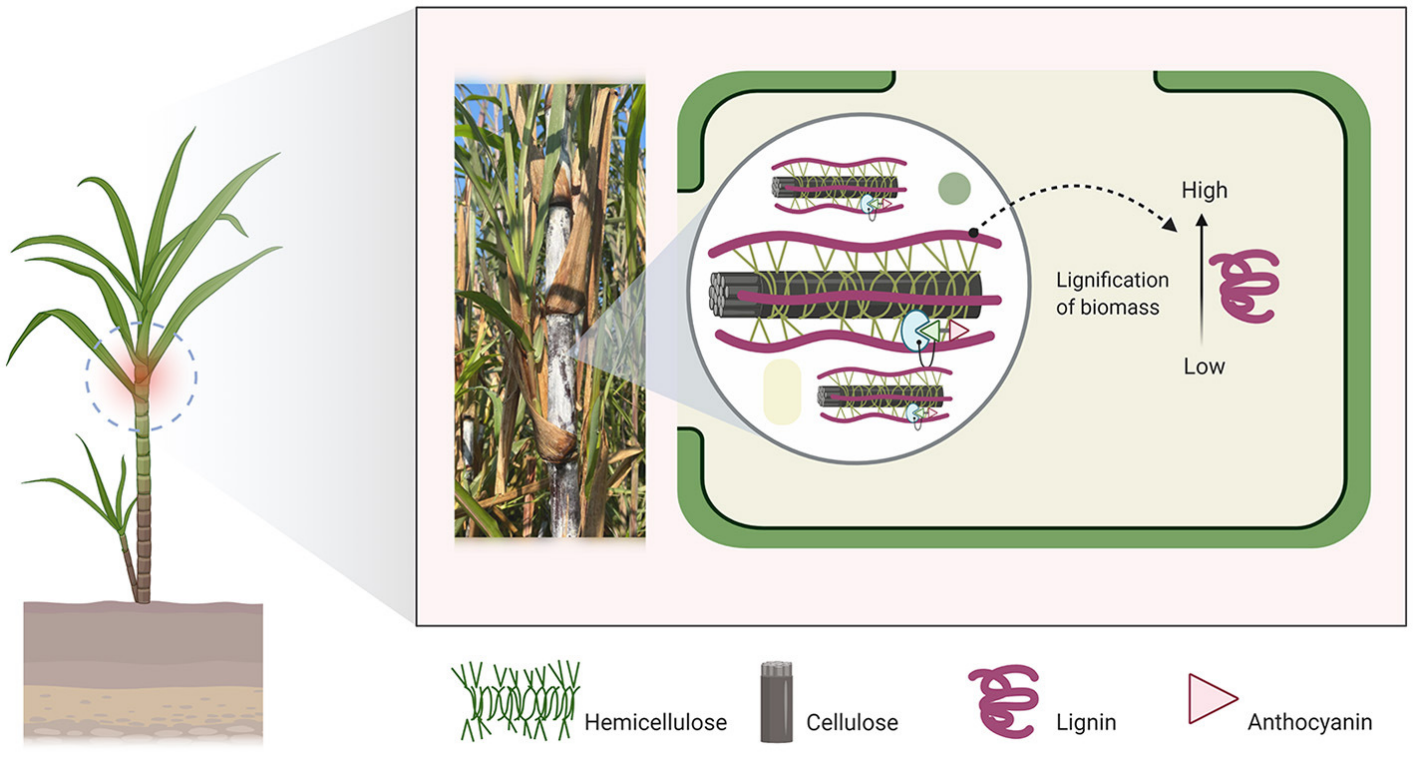
Black cane (BC: *Saccharum sinensis Robx*.) is a fast-growing grass (Poaceae) endemic to mainland Southeast Asia. It is a viable ruminant feedstock owing to its high concentration of soluble carbohydrates and anthocyanins. However, it needs pretreatment prior to ensiling due to the high amount of lignocellulose agricultural biomass.

Black cane (BC: *Saccharum sinensis* Robx.; Figure 1), often known as noble cane, is a complex interspecific hybrid of *Saccharum spontaneum* and *Saccharum officinarum*, a fast-growing grass (Poaceae) native to mainland Southeast Asia (1). Despite its stiffer stalks than sugarcane (*Saccharum officinarum*), BC was often employed in sugar manufacturing because of its high sucrose concentration. For instance, BC has recently gained recognition in Thailand for its quick growth, high fiber content, abundant protein content, and high anthocyanin content. All of these are compelling arguments for cultivating BC and considering it as a source of roughage for ruminant animals. Notably, anthocyanin's functional characteristics as a beneficial coloring molecule have been investigated in cattle; it controls heat stress relief, increases antioxidant activity, and stimulates the rumen microbiota (2, 3). As a result, it has the potential to enhance the quality of rumen-derived products (4). However, input from farmers suggests that feeding BC alone cannot entirely cover the production requirements of ruminants because of the poor palatability of anthocyanin and low dry matter intake. Generally, anthocyanins in plants have a bitter taste, and certain species, particularly *sinensis*, have a high concentration of lignocellulosic components (5). This has a negative impact on the availability of soluble carbohydrates during rumen digestion (6). It is therefore underutilized and undertreated by the majority of farmers. It is thus either left to rot in the field or burned in the open, both of which have substantial environmental consequences. For this reason, ensiling has been recommended as a viable means of preserving roughage and so supplying animals with a nutritious feed source (7).

Pretreatment of lignocellulose agricultural biomass or materials with inorganic salts prior to ensiling production has been identified in previous research as a viable strategy for recovering the nutritional content of highly lignified materials (8–10). Pretreatment with inorganic salts serves the overall purpose of increasing the efficiency with which hemicellulose and cellulose in biomass are hydrolyzed during fermentation. To accelerate the breakdown of hemicellulose and cellulose sugars in agricultural biomass, several inorganic salts such as sodium chloride, potassium chloride, calcium chloride, magnesium chloride, ferrous chloride, ferrous sulfate, iron (III) chloride and ferric sulfate were utilized as catalysts. Ferric sulfate was shown to improve the pretreatment efficiency of hemicellulose- and cellulose-rich materials at lower thresholds (8). The positive qualities of ferrous sulfate as a source of iron and for improving enzymatic hydrolysis and fermentation were investigated in that study largely *via* biomass biorefining. In general, ferrous sulfate is a kind of iron (Fe) and is a safe addition to feedstuffs. Iron is a key trace element required for animal health and productivity. In a previous study, increased ferrous sulfate monohydrate in diets (up to 1,000 mg Fe/kg) showed no adverse effect on growth performance in sheep, but improved nutrient digestibility, blood iron measurements, rumen fermentation, and bacterial populations (11). The recent FEEDAP panel concluded that 450 mg Fe/kg complete feed (ferrous sulfate monohydrate and/or ferrous sulfate heptahydrate formed in powder) with a moisture content of 12% is safe for bovines (12). However, no comparable research on the use of ferrous sulfate heptahydrate (FS) supplementation in small ruminants, such as goats, is available.

The use of iron supplements such as FS as silage additives may be an innovative strategy. However, the influence of FS on hemicellulose and cellulose sugar breakdown as well as the function of silage quality in ruminant performance in response to dietary ensiling have received little attention. In this study, the present study hypothesized that ensiled BC silages containing FS will improve ensiling properties, anthocyanin stability, rumen fermentation profile, and microbial populations. Therefore, the present study aimed to assess the effects of FS on fermentation quality, anthocyanin stability, ruminal biogases, rumen fermentation profile, and the microbial community of BC silages. The results from the study may provide an insight into the nature of the silage generation process and the importance of incorporating additives such as FS at the appropriate concentration for optimization. To date, references to BC's performance are fairly scanty. As a consequence, BC's pre-ensiled form or material was used as a negative control.

## Materials and Methods

### Silage Preparation

BC was developed and produced on a 500 m^2^ plot with a predetermined plot size of 75 × 75 cm at the Suranaree University of Technology (SUT) goat and sheep research farm in Nakhon Ratchasima Province, Thailand (14°52'49.1“N, 102°00'14.9”E, 243 m above sea level). An experimental field study was conducted from August 2017 to January 2018 during the monsoon season. Basal dressings of N:P:K (50:50:50; Hydro Thai Limited, Bang Kruai, Thailand) and goat manure were applied at 150 and 6,250 kg/ha, respectively, with urea (46:0:0) split administered at 30 kg/ha. Fresh BC was sampled on the 60th d following 120 d of regrowth by cutting well above the soil surface (10 cm above ground level). The fresh materials were sampled six times from random spots in the field. The obtained materials were then chopped with a crop cutter to a length of 2–3 cm and homogenized well. The biomass from each plot was kept separate for the ensiling trial. As a negative control, 1,000 g of chopped BC from each plot was stored at −20°C in sealed plastic bags (untreated or pre-ensiled material, *n* = 6). Similarly, 1,000 g of each ensiling material was treated with commercial ferrous sulfate heptahydrate (FS, FeSO_4_· × H_2_O; Merck KGaA, Darmstadt, Germany) at 0, 0.015, and 0.030% fresh matter separately for the biomass from each plot. Pre-ensiled and silage materials were evaluated; thereby four experimental treatments were pre-ensiled materials (FBC); ensiled FBC + 0% FS (SZF); ensiled FBC + 0.015% FS (SLF); ensiled FBC + 0.030% FS (SHF). The dosage level was chosen based on past research and animal safety assessments (11, 12). The additives were sprayed onto 1 kg of ensiling material and mixed thoroughly. Each fixed treatment was then vacuum sealed into a silo polyethylene bag (Hiryu KN type, Asahi Kasei Pax Corp., Tokyo). Each treatment was carried out in six silos. Ensiling took place for 21 d in the dark at 15–25°C. At the silo opening, the treated silage was sampled and homogeneously blended. Subsamples of BC silages and FBC were compared for evaluation of chemical and anthocyanin compositions, microorganisms, and fermentation quality.

### Chemical and Anthocyanin Compositions, Microorganisms, and Fermentation Quality

Each of six replications of pre-ensiled materials and silage materials at silo opening were divided into three subsamples. The first subsample was oven-dried at 55°C for 24 h to a consistent weight (about 2.0 g), air equilibrated, and then ground with a mesh size of 1 mm (Retsch SM 100 mill; Retsch Gmbh, Haan, Germany). The ground samples were evaluated for dry matter (DM; AOAC#934.01), ash (AOAC#942.05), and crude protein (CP; total N 6.25; AOAC#988.05) using the Association of Official Analytical Chemists procedures (13). The organic-matter content (OM) was calculated as 100% minus the ash percentage obtained after 5 h of incineration in a muffle furnace at 550°C. The Van Soest et al. (14) technique was employed successively to identify neutral detergent fiber (NDF; with heat stable +/– amylase; ash included), acid detergent fiber (ADF; ash included), and acid detergent lignin (ADL). Hemicellulose content was determined as NDF minus ADF, while cellulose content was calculated as ADF minus ADL. The WSC content was determined using peak detection and high-performance liquid chromatography (HPLC), as reported in a recent work (15).

Furthermore, the second subsample was lyophilized, ground, and extracted at 50°C for 24 h with 0.01 N hydrochloric acid (HCl) dissolved in an 80% methanol solution, and the supernatant was collected and transferred into a 50-mL volumetric flask for HPLC determination of anthocyanin composition (16–18). The remaining silage substrates were utilized to evaluate fermentative quality by extracting silages as previously reported (17) with a modest modification. Forty-gram amounts of each silage were put in a 500-mL beaker, filled with 200 mL of distilled water, and mixed for 30 mins at 27–28°C. Filter paper (WhatmanTM No. 1441-125, GE Healthcare Life Sciences, Marlborough, MA, USA) was used to strain the mixture. Following that, the pH was measured using a portable pH meter (Oakton pH 700, Long Branch, New Jersey, NJ, USA). Lactic acid and VFAs were measured using HPLC (Agilent Technologies 1260 Infinity, Santa Clara, CA, USA) according to a previously established technique (19), with peak detection compared and computed as stated by Purba et al. (20). Ammonia nitrogen (NH_3_-N) was measured using a spectrophotometer (Varioskan-LUX multimode microplate reader, Thermo Scientific, Waltham, MA, USA) according to the previously reported procedures (21, 22).

The final subsample was utilized for microorganism count by creating pre-ensiled materials and silage forms using the plate count technique (23) with a slight modification. In a 500-mL beaker, about 20 g of subsample was covered with 180 mL sterilized distilled water, and beakers were shaken vigorously at 30°C for 2–3 h on a rotary shaker at 200 rpm; successive dilutions (10^−1^-10^−5^) were conducted correctly in sterilized distilled water. Without bubbles, a volume of 20 μL of each created dilution was poured and placed on agar plates. The plates were then incubated for 48 h in an anaerobic jar at 30°C. For microbiological assessment, incubated plates of de Man, Rogosa, and Sharpe (MRS) agar (Himedia, Mumbai, India) were used to count the number of lactic acid bacteria; incubated plates of blue light broth agar (Sigma-Aldrich, Gillingham, UK) were used to count the number of coliform bacteria; incubated plates of nutrition agar (Difco, St. Louis, MO, USA) were used to count the number of aerobic bacteria; and incubated plates of potato dextrose agar (Titan Biotech, Rajasthan, India) were used to count the number of yeasts and molds. The yeasts were distinguished from molds and bacteria using techniques in colony appearance and cell shape.

### *In vitro* Ruminal Fermentation

The incubation fluid was a 1:2 (v/v) mixture of rumen fluid and artificial buffer solution. Rumen fluid was collected from three Thai-native Anglo-Nubian male goats (20.2 ± 1.6 kg live weight) before morning feeding using a stomach tube attached to a manual pump (6, 24). The experimental procedure was authorized by Suranaree University of Technology Animal Ethics Committee (SUT 4/2558); animal health care was consistent with our earlier work (25). The goats were given a total mixed ration containing cassava hay (470 g/kg DM), rice stubble (350 g/kg DM), urea (30 g/kg DM), and constant components (150 g/kg DM) at 07:00 and 15:00 h and had full access to drinking water. Constant component components were cassava pulp (7.5% DM), cassava chip (39% DM), mineral mix (1.6% DM), palm meal (28% DM), premix (2.4% DM), rice bran (11% DM), soybean meal (8% DM), sulfur (2.5% DM), and sunflower oil (2% DM). In this study, the rumen fluid combination was filtered through four layers of nylon (400 μm; Fisher Scientific S.L., Madrid, Spain), mixed with buffer solution, and maintained at 39°C in a water bath while being constantly flushed with CO_2_. Anaerobic buffer solution was created in the same manner as stated in the methodology for *in vitro* gas production (26).

In order to gain a better understanding of how silage quality affects ruminant performance in response to dietary ensiling, *in vitro* ruminal fermentation was performed in this study. A 100 mL calibrated Hohenheim glass syringe (Toitu, TOP Surgical Manufacturing Co., Ltd., Tokyo, Japan) was filled with 500 mg of dry pre-ensiled materials and silage materials and incubated in a water bath shaker (39°C) for 24 h with 30 mL of rumen fluids-buffer mixture. Within the incubation, 4 treatments or incubated materials were evaluated in 18 repetitions, and the incubation was repeated 3 periods (3 runs). For each run, three syringes were made and supplied as blanks (rumen fluids–buffer mixture alone). After 6, 12, and 24 h of incubation, the gas volume was measured. The cumulative volume of gas production was calculated using the fitted model of Orskov and Mcdonald (27), with the equation: *y* = *a* + *b* (1^−*e*(−*ct*)^), where a (mL/g DM) is gas production from the soluble fraction, b is gas production from the insoluble fraction (mL/g DM), c (/h) is the gas production rate constant for the insoluble fraction (*b*), *t* (h) is the incubation time, (*a* + *b*) (mL/g DM) is the potential gas production, and *y* is the gas produced at time *t* (mL/g DM).

### Analysis of Fermentation Characteristics, DNA Extraction, and Quantitative Real-Time PCR (QPCR)

Methane and carbon dioxide gases, fermentation end products, and microbiological analyses were all taken from six replicated glass syringes during the gas yield analysis. This was carried out for incubation times of 6, 12, and 24 h, respectively. In order to quantify the amounts of methane and carbon dioxide, 10 mL of gas was transferred into a disposal syringe with three-way stopcocks (Agilent 7890A, Agilent Technologies). Calibration and chromatographic conditions were previously published (28–30). When the glass plungers of the syringes were opened, the pH was instantly measured using a pH meter (Oakton 700, Cole-Parmer, Vernon Hills, IL). A volume of 10 mL of rumen fluids was generated for measuring anthocyanin, ammonia nitrogen, and volatile fatty acids (VFAs) and examined in a way similar to the procedure described above for pre-ensiled materials and silage forms. The remaining contents of each culture syringe were collected for microbiological detection using a quantitative real-time PCR (qPCR) reaction.

Each culture sample was harvested for genomic DNA using the technique outlined by Yu and Morrison (31). Total genomic DNA was extracted using the QIAamp DNA Stool Mini Kit (Qiagen, Hilden, Germany) from 1 mL of homogenized rumen fluid. DNA yield was assessed using the NanoDrop NanoVue spectrophotometer (GE Healthcare Bio-Sciences, Pittsburgh, PA, USA) at an absorbance ratio of 260:280. To protect the DNA, it was eluted with suitable dilutions (volume of nuclease-free water) and kept at −20°C until further analysis.

Quantification of the relative abundances of selected primers in genomic DNA extracted from rumen fluids was performed using a QuantiTect SYBR Green RT–PCR Kit (full master mix; Qiagen) equipped with the selected primer set and a Roche Lightcycler 480-II (Roche Applied Science, Basel, Switzerland), using the previously described amplification and qPCR settings (32). References for the relative abundance of total bacteria, *Ruminococcus albus, Ruminococcus flavefaciens, Fibrobacter succinogenes, Butyrivibrio fibrisolvens, Megasphaera elsdenii, Streptococus bovis*, Methanogen, and protozoa were purchased from Vivantis Technologies Sdn Bhd (Selangor Darul Ehsan, Malaysia) for use as a guide for the experiment [Supplementary Table 1; (33–35)]. Prior to performing qPCR experiments, a sixfold serial dilution of pooled DNA was generated to establish a standard curve. To ensure reproducibility, the qPCR assays for each chosen species or group of bacteria were carried out in triplicate using both standards and genomic DNA samples. The LightCycler 480 software version 1.2.9.11 was used to transform the Ct data into normalized relative numbers, compensated for PCR efficiency (Roche Applied Science). The absolute abundances of each chosen species or group of microorganisms were represented as rrs gene copies/mL of culture materials.

### Statistical Analysis

The average of data for silage fermentation quality from 6 prepared silos, were processed as a completely randomized design and submitted to one-way ANOVA using the MIXED procedure of SAS 9.4. The statistical model used was: *Y*_*ij*_ = μ + *B*_*i*_ + *i*_*j*_, where *Y*_*ij*_ is the observation, μ is the overall mean, *B*_*i*_ is the BC forms or substrates (_*i*_ = 1–4, FBC, SZF, SLF, SHF), and *i*_*j*_ is the error. The results are shown as mean values and standard errors of the means. Orthogonal polynomials were used to analyze the effects of FS addition as well as the comparison of pre-ensiled vs. ensiled materials. The significance level was set at *P* < 0.05, which represents statistically significant differences.

The average of data for anthocyanin stability observed at 6, 12, and 24 h of post-incubation from 3 incubated runs were analyzed using the MIXED procedure in SAS 9.4 as a completely randomized design with repeated measurements. The following statistical model was used: *Y*_*ijkl*_ = μ + *B*_*i*_ + *T*_*j*_ + *C*_*k*_ (*B*) + (*B* × *T*)_*ij*_ + ε_*ijkl*_, where *Y*_*ijkl*_ is the observation, μ is the overall mean, *B*_*i*_ is the fixed BC form effect or substrate (_*i*_ = 1–4, FBC, SZF, SLF, SHF), *T*_*j*_ is the fixed effect of the sampling time (_*j*_ = 1–3, post-incubation at 6, 12, and 24 h), *C*_*k*_ is the random effect of the rumen fluid nested within the period of observation (_*k*_ = 1–3, run 1, 2, and 3), (*B* × *T*)_*ij*_ is the interaction between substrate used and sampling time, and ε_*ijkl*_ is the residual error. The compound symmetry of the covariance structure was verified using Akaike's information criterion in SAS's mixed model. To determine if the data were normally distributed, the Kolmogorov–Smirnov test was utilized. According to the repeated measures design, the statistical significance of the substrate effect was assessed against the variance of the gas syringe nested inside the substrate used. Tukey's HSD test was performed to determine which factors had a different influence on the dependent variable. Significance was defined as *P* < 0.05, and the trend was defined as 0.05 < *P* <0.10.

Because data from three consecutive runs of incubation and data collected at 6, 12, and 24 h post-incubation were comparable, data for ruminal biogases, rumen fermentation profile, and microbial community observed at 6, 12, and 24 h of post-incubation from 3 incubated runs were processed as a completely randomized design and submitted to one-way ANOVA using the MIXED procedure of SAS 9.4. The statistical model used was: *Y*_*ij*_ = μ + *B*_*i*_ + *i*_*j*_, where *Y*_*ij*_ is the observation, μ is the overall mean, *B*_*i*_ is the BC forms or substrates (_i_ = 1 to 4, FBC, SZF, SLF, SHF), and *i*_*j*_ is the error. The results are presented in the form of mean values and standard errors of the means. The effects of FS addition were analyzed using orthogonal polynomials, as well as the comparison of pre-ensiled vs. ensiled materials. The significance level was set at *P* > 0.05, which represents statistically significant differences.

## Results

### Pre-ensiled Black Cane Chemical Composition, Anthocyanin Content, and Microorganism Count

The chemical compositions of black cane (BC) pre-ensiled materials, anthocyanin concentrations, and pH values are listed in Table 1. The DM of BC prior to ensiling was 15.28%, and OM, CP, NDF, ADF, and ADL were 88.65, 6.54, 51.17, 31.71, and 3.78% DM, respectively. The HC, CEL, and WSC of BC were 194.61, 279.33, and 26.17 g/kg DM, respectively. Additionally, BC had a high concentration of anthocyanin at a slightly acidic pH: 4.28% cyanidin-3-glucoside (C3G), 8.49% pelargonidin-3-glucoside (P3G), 7.23% delphinidin (Del), 12.52% peonidin-3-O-glucoside (Peo3G), 10.44% malvidin-3-O-glucoside (M3G), 14.25% cyanidin (Cya), 7.23% pelargonidin (Pel), and 35.55% malvidin (Mal) of total anthocyanin (Table 1). However, pre-ensiled BC appeared to have a low lactic acid bacteria content (2.42 × 10^4^ CFU/g FM), with undesirable ensiling microorganisms such as coliform bacteria, aerobic bacteria, and yeast predominating (Table 2).

**Table 1:** Chemical composition, anthocyanin content, and pH value of black cane prior to ensiling (pre-ensiled materials) and silages treated with ferrous sulfate heptahydrate after 21 d.

**Item^**1**^**	**Treatment**				**SEM**	***P*** **-value**		
	**FBC**	**SZF**	**SLF**	**SHF**		**T**	**L**	**Q**
**Chemical composition**
DM, g/kg FM	152.80^d^	153.97^c^	154.57^b^	159.81^a^	0.192	0.003	<0.001	<0.001
OM, g/kg DM	886.52^a^	885.93^a^	881.08^b^	874.61^c^	0.220	<0.001	0.021	<0.001
CP, g/kg DM	65.38	64.79	64.66	65.31	0.323	0.414	0.836	0.253
NDF, g/kg DM	511.74	510.78	509.59	509.09	0.714	0.082	0.124	0.918
ADF, g/kg DM	317.13	315.58	313.52	313.69	0.763	0.063	0.087	0.551
ADL, g/kg DM	37.80^a^	39.38^a^	27.47^b^	25.29^c^	0.301	<0.001	<0.001	0.009
HC, g/kg DM	194.61	195.20	196.07	195.41	0.363	0.130	0.092	0.222
CEL, g/kg DM	279.33^c^	276.19^c^	286.05^b^	288.39^a^	0.663	<0.001	<0.001	0.002
WSC, g/kg DM	26.17^a^	10.32^d^	11.67^c^	13.58^b^	0.145	<0.001	<0.001	<0.001
**Anthocyanin content**
Total anthocyanin, mg/g DM	0.926^a^	0.245^d^	0.339^c^	0.403^b^	0.007	<0.001	<0.001	<0.001
C3G, mg/g DM	0.047^a^	0.014^b^	0.015^b^	0.015^b^	0.003	0.001	<0.001	0.003
P3G, mg/g DM	0.093^a^	0.021^b^	0.026^b^	0.027^b^	0.008	0.002	<0.001	0.002
Del, mg/g DM	0.079^a^	0.049^c^	0.060^a^	0.071^a^	0.004	0.012	0.634	0.005
Peo3G, mg/g DM	0.137^a^	0.047^c^	0.058^c^	0.071^b^	0.009	0.001	0.003	0.004
M3G, mg/g DM	0.114^a^	0.041^d^	0.079^b^	0.088^b^	0.003	<0.001	0.033	<0.001
Cya, mg/g DM	0.156^a^	0.073^b^	0.076^b^	0.080^b^	0.012	0.004	0.005	0.026
Pel, mg/g DM	0.079^a^	0.005^b^	0.005^b^	0.007^b^	0.011	0.005	0.004	0.044
Mal, mg/g DM	0.389^a^	0.040^c^	0.081^b^	0.118^b^	0.006	<0.001	<0.001	<0.001
pH value	5.47^a^	4.81^b^	4.73^b^	4.25^c^	0.179	0.002	0.001	0.467

*^1^ DM, dry matter; FM, fresh matter; OM, organic matter; CP, crude protein; NDF, neutral detergent fiber; ADF, acid detergent fiber; ADL, acid detergent lignin; HC, hemicellulose; CEL, cellulose; WSC, water-soluble carbohydrate; C3G, cyanidin-3-glucoside; P3G, pelargonidin-3-glucoside; Del, delphinidin; Peo3G, peonidin-3-O-glucoside; M3G, malvidin-3-O-glucoside; Cya, cyanidin; Pel, pelargonidin; Mal, malvidin; FBC, pre-ensiled materials; SZF, ensiled FBC + 0% ferrous sulfate heptahydrate; SLF, ensiled FBC + 0.015% ferrous sulfate heptahydrate; SHF, ensiled FBC + 0.030% ferrous sulfate heptahydrate; SEM, standard error of mean. T, effect of pre-ensiled vs. ensiled materials; L, linear effect of ferrous sulfate heptahydrate additions; Q, quadratic effect of ferrous sulfate heptahydrate additions*.

*^a−d^Means with a different superscript in the same row differ significantly (P <0.05)*.

**Table 2:** Microbiological count and organic component concentration of black cane prior to ensiling (pre-ensiled materials) and silages treated with ferrous sulfate heptahydrate after 21 d.

**Item^**1**^**	**Treatment**				**SEM**	***P*** **-value**		
	**FBC**	**SZF**	**SLF**	**SHF**		**T**	**L**	**Q**
**Microorganism, CFU/g FM**
Lactic acid bacteria	2.42 × 10^4d^	5.35 × 10^7c^	8.48 × 10^7b^	1.11 × 10^8a^	0.348	<0.001	<0.001	<0.001
Coliform bacteria	2.22 × 10^7a^	2.53 × 10^4b^	2.22 × 10^4c^	2.12 × 10^4d^	0.209	<0.001	<0.001	<0.001
Aerobic bacteria	4.55 × 10^6a^	8.89 × 10^5b^	2.02 × 10^5c^	6.36 × 10^4d^	0.214	<0.001	<0.001	<0.001
Yeasts	2.63 × 10^8a^	4.14 × 10^6b^	nd	nd	0.161	<0.001	<0.001	<0.001
Molds	nd	nd	nd	nd	0.000	0.000	0.000	0.000
**Organic compound, g/kg DM**
Lactic acid	nd	34.81^c^	42.94^b^	47.86^a^	0.231	<0.001	<0.001	<0.001
Acetic acid	nd	26.44^c^	28.00^b^	29.36^a^	0.222	<0.001	<0.001	<0.001
Propionic acid	nd	nd	nd	nd	0.000	0.000	0.000	0.000
Butyric acid	nd	nd	nd	nd	0.000	0.000	0.000	0.000
Ammonia nitrogen	nd	0.31	0.27	0.25	0.198	0.792	0.514	0.745

*^1^ DM, dry matter; CFU, colony-forming unit; FM, fresh matter; nd, not detected; FBC, pre-ensiled materials; SZF, ensiled FBC + 0% ferrous sulfate heptahydrate; SLF, ensiled FBC + 0.015% ferrous sulfate heptahydrate; SHF, ensiled FBC + 0.030% ferrous sulfate heptahydrate; SEM, standard error of mean. T, effect of pre-ensiled vs. ensiled materials; L, linear effect of ferrous sulfate heptahydrate additions; Q, quadratic effect of ferrous sulfate heptahydrate additions*.

*^a−d^ Means with a different superscript in the same row differ significantly (P < 0.05)*.

### Black Cane Chemical Composition, Anthocyanin Content, and Ensiling Characteristics

BC pre-ensiled materials and silages treated with ferrous sulfate heptahydrate (FS) after 21 d exhibited different chemical compositions, anthocyanin concentration, and pH value (Table 1). Increasing the dilution concentrations of FS incorporated into silages was observed to linearly decrease DM, anthocyanin, and nutritive value losses (*P* < 0.001). The CP content of the BC silages was not affected (*P* > 0.253) by increasing the dilution concentrations of FS to the BC silages. However, the lignin values decreased linearly as the percentage of FS increased up to 0.030%. Compared to other FS-treated silages, BC silage treated with 0.030% FS dilution appeared to have optimum biomass conservation of cellulose content during ensilage. The WSC also linearly increased (*P* < 0.001) with increasing the dilution concentrations of FS incorporated into silages (Table 1).

In comparison to pre-ensiled BC, 21 d of ensilage resulted in a positive effect on pH values; all BC silages had a pH range of 4.25–4.81 (Table 1; *P* = 0.002). These desirable pH values resulted in increased lactic acid bacteria and decreased coliform bacteria, aerobic bacteria, and yeast content in all BC silages (Table 2; *P* < 0.001). As expected, increasing the dilution concentrations of FS added to silages increased the lactic acid bacteria content linearly (*P* < 0.001) and decreased the coliform bacteria, aerobic bacteria, and yeast content of all BC silages (*P* < 0.001). Despite the fact that the ammonia content remained unchanged after ensiling BC silages, the levels of lactic acid and acetic acid increased linearly as the dilution concentration of FS incorporated into silage was increased (*P* < 0.001). Mold, propionic acid, and butyric acid were also undetectable in all BC silages.

### Anthocyanin Stability of Incubated Black Cane

**Figure 2:**
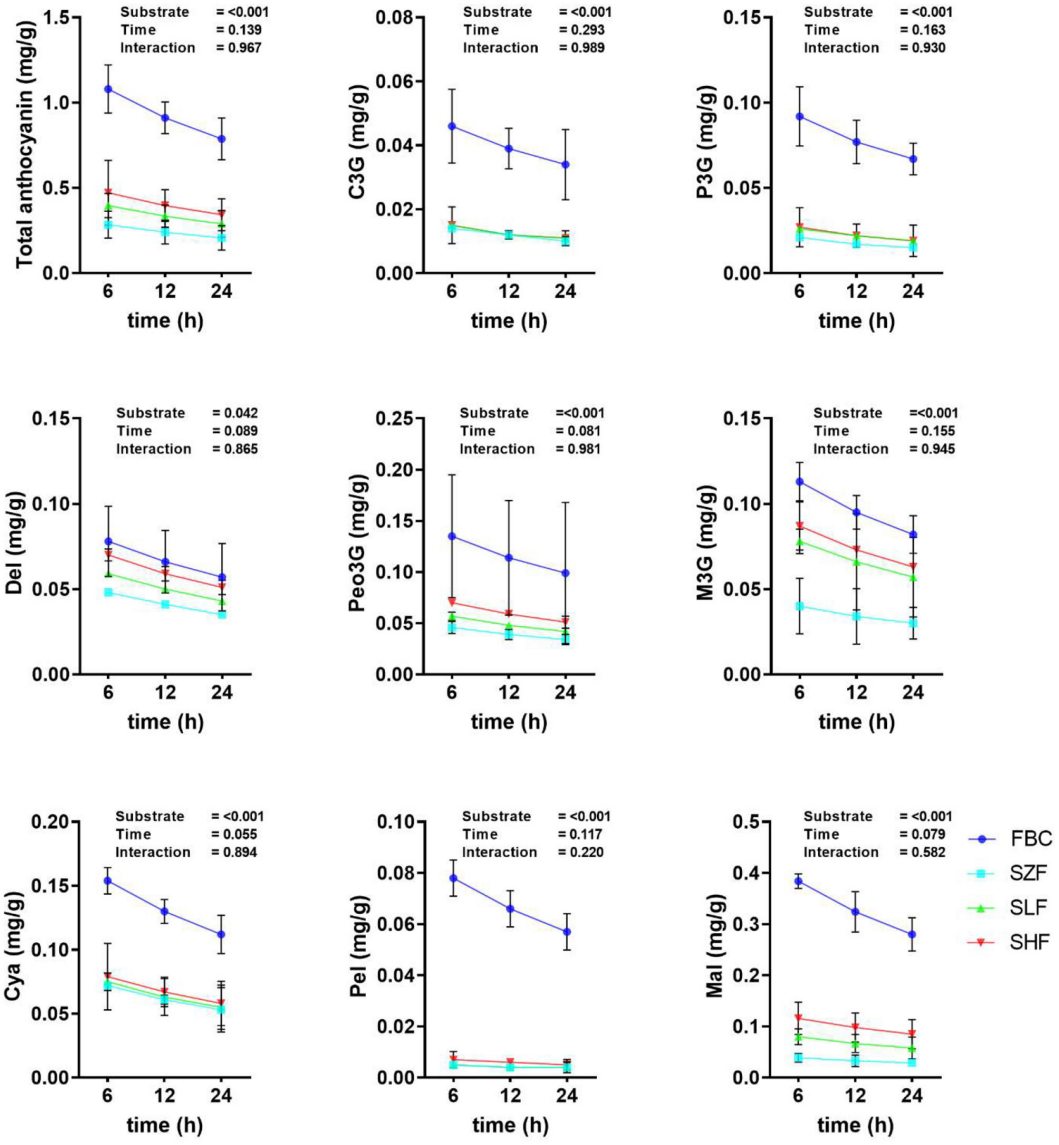
The anthocyanin concentration of black cane prior to ensiling (pre-ensiled materials) and silages treated with ferrous sulfate heptahydrate, at three time points (6, 12, 24 h) during the *in vitro* rumen incubation (means ± SEM; *n* = 18). C3G, cyanidin-3-glucoside; P3G, pelargonidin-3-glucoside; Del, delphinidin; Peo3G, peonidin-3-O-glucoside; M3G, malvidin-3-O-glucoside; Cya, cyanidin; Pel, pelargonidin; Mal, malvidin; FBC, pre-ensiled materials; SZF, ensiled FBC + 0% ferrous sulfate heptahydrate; SLF, ensiled FBC + 0.015% ferrous sulfate heptahydrate; SHF, ensiled FBC + 0.030% ferrous sulfate heptahydrate. Overall, substrate effect, *P* < 0.05; incubation time, *P* < 0.05; interaction, *P* < 0.05.

To determine the stability of anthocyanins in rumen fluid, we quantified anthocyanins in pre-ensiled BC materials and silages at three time points (6, 12, and 24 h) during *in vitro* rumen incubation (Figure 2). After 24 h of ruminal fermentation, the anthocyanin content of BC remained constant. There was no interaction between the substrate used and the incubation time (*P* > 0.10). Notably, prior to *in vitro* rumen incubation, each concentration of anthocyanin in BC pre-ensiled materials and silages has been reported (Table 1). While there were some minor discrepancies between the values in Table 1 and Figure 2, their magnitudes were quite similar.

### *In vitro* Rumen Fermentation Profile and Ruminal Biogases of Incubated Black Cane

**Figure 3:**
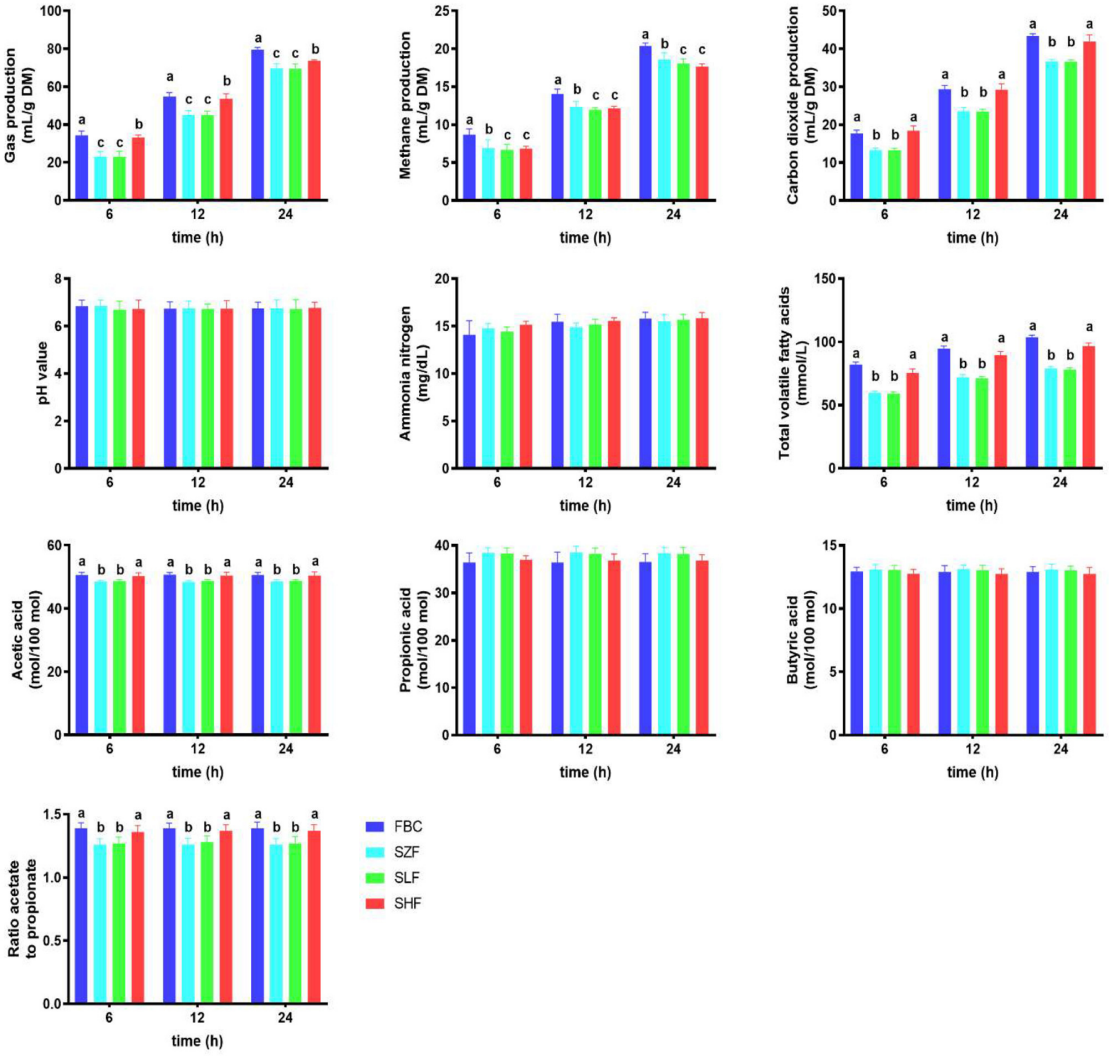
Selected ruminal biogas of black cane prior to ensiling (pre-ensiled materials) and silages treated with ferrous sulfate heptahydrate, at three time points (6, 12, 24 h) during the *in vitro* rumen incubation (means ± SEM; *n* = 18). FBC, pre-ensiled materials; SZF, ensiled FBC + 0% ferrous sulfate heptahydrate; SLF, ensiled FBC + 0.015% ferrous sulfate heptahydrate; SHF, ensiled FBC + 0.030% ferrous sulfate heptahydrate. ^a−*c*^ A different superscript in similar sampling time indicates a significantly different mean (*P* < 0.05).

After 24 h of ruminal fermentation, the concentrations of ammonia nitrogen and pH of incubated pre-ensiled materials (FBC) and FS-treated silages (SZF, SLF, and SHF) were comparable, but not the concentrations of total VFAs (Figure 3). Increased dilution concentrations of FS in silages as providing substrate incubation consistently resulted in a decrease in total VFAs at 6, 12, and 24 h post-incubation (*P* < 0.05), but not for the SHF (0.030% FS-treated silage). We noticed that FBC and SHF consistently had higher VFA concentrations than SZF and SLF at 6, 12, and 24 h after post-incubation (*P* < 0.05). Acetic acid concentrations were also observed to be higher in this circumstance (*P* < 0.05). However, increasing the dilution concentrations of FS to the BC silages had no effect on the concentrations of propionic acid or butyric acid (Figure 3; *P* > 0.05).

As illustrated in Figure 3, residual FS in BC influenced ruminal biogases 24 h after incubation. Incubation of SZF (0% FS-treated silage) and SLF (0.015% FS-treated silage) as providing substrate incubation resulted in a greater decline in carbon dioxide production (*P* < 0.05), whereas incubation of SHF (0.030% FS-treated silage) resulted in a positive trend similar to that of FBC, releasing carbon dioxide for 24 h. Nonetheless, increasing the dilution concentrations of FS incorporated into silages as providing substrate incubation was found to linearly reduce methane production after 24 h post-incubation (*P* < 0.05). Among the FS-treated silages incubated, SHF had the highest total production for 24 h, but it was still lower than FBC.

### Microbial Community of Incubated Black Cane

**Figure 4:**
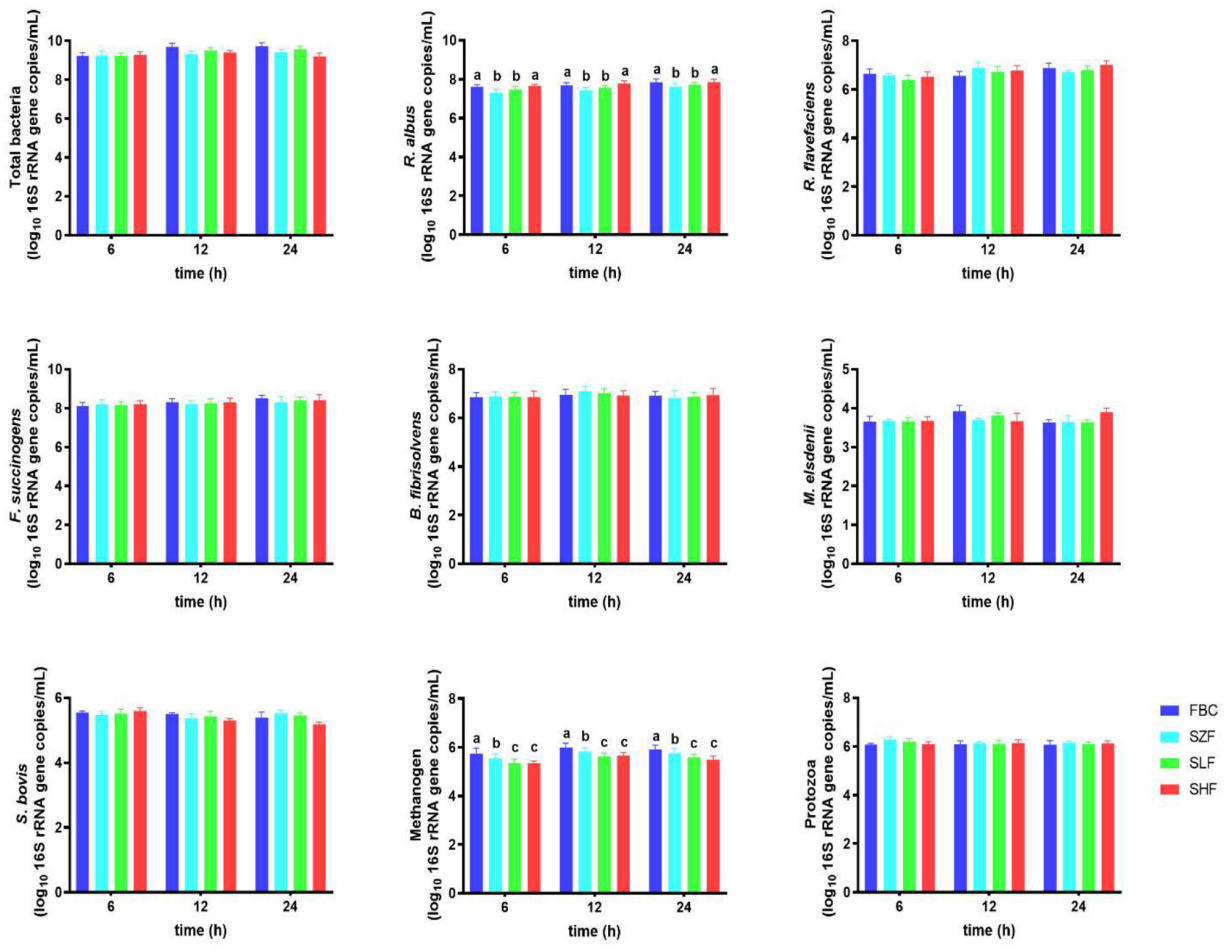
Microbial population of black cane prior to ensiling (pre-ensiled materials) and silages treated with ferrous sulfate heptahydrate, at three time points (6, 12, 24 h) during the *in vitro* rumen incubation (means ± SEM; *n* = 18). FBC, pre-ensiled materials; SZF, ensiled FBC + 0% ferrous sulfate heptahydrate; SLF, ensiled FBC + 0.015% ferrous sulfate heptahydrate; SHF, ensiled FBC + 0.030% ferrous sulfate heptahydrate. ^a−*c*^A different superscript in similar sampling time indicates a significantly different mean (*P* < 0.05).

After 24 h of ruminal fermentation, the major microbial communities, including *R. flavefaciens, F. succinogenes, B. fibrisolvens, M. elsdenii, S. bovis*, and protozoa, from incubated pre-ensiled materials (FBC) and FS-treated silages (SZF, SLF, and SHF) as providing substrate incubation, were similar, but not the *R. albus* and methanogen (Figure 4). Incubation of SZF (0% FS-treated silage) and SLF (0.015% FS-treated silage) led to a greater decrease in *R. albus* abundance (*P* < 0.05), whereas SHF (0.030% FS-treated silage) resulted in a positive trend similar to that of FBC, increasing *R. albus* abundance in rumen incubation for 24 h. Additionally, increasing the dilution concentrations of FS in silages was found to linearly decrease the abundance of methanogen in rumen incubation for 24 h (*P* < 0.05).

## Discussion

### Black Cane Has a High Proportion of Moisture, Fiber, Anthocyanin, and Lignin, as Well as Undesirable Ensiling Microorganisms

To the best of our knowledge, the chemical makeup of black cane (BC, *S. sinensis*) has not yet been determined in its entirety. Our data indicate that BC has a greater fiber content than other tropical roughages or grasses, which may make it a useful carbohydrate-rich nutritional feed for ruminant animals (36). More intriguingly, the current investigation discovered that BC has a lot of anthocyanin. Anthocyanins are naturally occurring pigments found in a broad variety of plants. They are not only employed as a food colorant addition, but they can also be utilized to reduce the risk of numerous illnesses and have several functional and biological properties for ruminants, such as antioxidant activity (4, 7). Within a slightly acidic pH range, the overall anthocyanin and anthocyanin levels were comparable to those found in Chinese sugarcane (16, 37), but particular anthocyanin amounts, such as cyanidin-3-glucoside (C3G), pelargonidin-3-glucoside (P3G), delphinidin (Del), peonidin-3-O-glucoside (Peo3G), malvidin-3-O-glucoside (M3G), cyanidin (Cya), pelargonidin (Pel), and malvidin (Mal) were higher in BC. However, it should be noted that BC in the current research was shown to have a significant level of lignification or silicification of lignocellulosic biomass, resulting in a lower nutritional value. Silage fermentation or pretreatment of lignocellulose is therefore advised as a strategy to recover the nutritional value of BC (8–10), and it may be extended, at least qualitatively, to realistic feeding settings (6). Silage generated by the anticipated BC processing might potentially be used to mitigate some of the current environmental concerns. Despite an abundance of CP content (ranged of 6.5–7%), the current research also showed that the DM, OM, NDF, ADF, ADL, HC, CEL, and WSC BC pre-ensiled materials are generally adequate for additional roughage ensiling. This conclusion was reached because of FBC's (pre-ensiled BC materials) somewhat high ensilability index (EI; +12), which was utilized to anticipate the ensilage process (38). Despite this, FBC seems unable to develop microorganisms that facilitate the process of ensiling. The suggested experimental additives, including FS, may also be used to control the quantity of FBC microorganisms during silage fermentation, at either low FS (SLF) or high FS (SHF) levels.

### Ferrous Sulfate Heptahydrate Enhanced Black Cane Chemical Composition, Anthocyanin Content, and Ensiling Characteristics

The present results suggested that FS enhanced the amount of biodegradable lignocellulose in BC during ensiling. This was expected, since increasing the concentration of FS caused a change in the degraded organic content of BC during ensiling. These findings were supported by data from previous biorefining of lignocellulosic biomass study (8), which discovered that FS may act as a catalyst for the breakdown of hemicellulose and cellulose sugars in corn stover. In general, hemicellulose degrades faster than cellulose during ensiling. However, it was shown that FS effectively degraded cellulose in BC, resulting in a shift in the amount of non-structural and structural carbohydrates. The present results were congruent with those of Fly et al. (39), who discovered that hemicellulose remained constant as an impact on iron bioavailability in animals. A precise explanation is unknown; nevertheless, it is possible that the reason is related to certain cellulase activities in the FS pretreatment substrate. The metal ions in the FS ingredients may enhance the activity of cellulose rather than hemicellulose, increasing enzyme accessibility to cellulose and aiding cellulose enzymatic stability (8). Thus, the explanation for the rise in WSC after FS addition among ensiled materials in our study might be related to an increase in lignocellulosic component breakdown (5), resulting in better effects on non-structural carbohydrate and degradation of structural carbohydrate. It is probable that increasing the WSC content of BC silage resulted in a decrease in DM loss and energy recovery because of the homolactic acid bacteria's use of WSC (e.g., glucose) for development and generation of lactic acid during fermentation (40).

According to the findings of this study, BC ensiling seems to be beneficial when FS recovered more quickly to reduce DM loss in this research. It has been suggested that a successful application of silage production is dependent on the stability of ensiled materials, which have a higher recovery of DM, energy, and highly digestible nutrients than pre-ensiled materials (41). This might be accomplished by maintaining a constant moisture content throughout silage fermentation. Previously, it was hypothesized that moisture content may explain whether fermentation was good, average, or poor—excess moisture content resulted in a larger loss of DM content and low-quality silages (41). Nevertheless, adding FS to the BC ensiling process resulted in a slight reduction of OM. Our findings indicated that the use of FS for grass silage is incomplete and that additional preservatives or substrates should be provided. For instance, the most frequent method is utilizing molasses, which has the capacity to significantly limit the loss of chemical properties of ensiled materials throughout the preservation process by reducing both respiration and fermentation (42). More research is required to understand how FS and molasses work together to alter the lignocellulose chemical composition of agricultural biomass.

It is critical to investigate the quantitative changes in anthocyanin content during preservation in order to accurately provide anthocyanin to ruminant animals *via* ensiled BC. It is expected that most of the anthocyanin will be lost during silage fermentation. The present findings demonstrated that ensiling BC results in a decrease in anthocyanin, but adding FS to BC linearly reversed this nutrition loss. Hosoda et al. (43) reported that high pH and temperature as well exposure to oxygen and light promoted anthocyanin degradation. Our finding of higher silage pH coinciding with greater anthocyanin reduction is in line with that. Interestingly, more previous research (17, 19, 43) concluded that the rate of anthocyanin loss was positively correlated with the pH value under similar ensilage conditions. Following up on the findings of the present study, it was discovered that all silages had a lower pH value after 21 d of ensilage, indicating that FS provided a more favorable environment for the stability of anthocyanins. As a result, the range of anthocyanins in ensiled BC alone was reduced to a greater extent than the range of anthocyanins in ensiled BC treated with FS. This implied that FS was responsible for the decreased pH in FS-treated silages (4.73–4.25). Another possible explanation is that, as previously stated, FS may promote the breakdown of the lignin fraction in BC, increasing the availability of sugar and, as a result, promoting fermentation acid production. The current findings were consistent with previous research, which found that ~42% of anthocyanin was preserved in colored barley (19) and ~52% in anthocyanin-rich purple corn stover silage (17). Our findings also indicated that pH-increasing anthocyanin had a superior effect on all anthocyanins, with the exception of peonidin-3-O-glucoside BC, which was consistent with findings from previous studies (17, 44, 45).

To our knowledge, this is the first research to describe the systematic influence of FS on the quality and content of silage fermentation. The results of this study demonstrated that FS improved the ensiling properties of BC, including the microbiological count and the concentration of organic components. In all silages treated with FS, FS significantly increased the number of lactic acid bacteria and inhibited yeast and mold growth. These occurrences may be due to an acidic environment generated by the additament of FS, which promotes the growth of beneficial microbial silages and results in the production of more lactic acid and acetic acid in the silages rather than propionic acid and butyric acid. As previously stated, within the lowered pH as an FS effect on BC, our findings validated the evidence of Kung et al. (41) that the decreased pH may be linked to the concentration of buffering capacity and lactic acid. Thus, all improvements in lactic acid bacteria in this research may be due to adjusting the pH during the ensiling of BC. As is well known, lactic acid is the primary product of crop or grass preservation because of the capacity of lactic acid bacteria to exercise metabolic control over water availability for growth. Furthermore, results from the present study indicated that acetic acid levels were mild, suggesting that yeasts were not discovered in our silages during BC preservation with FS. Previously, it was thoroughly described that moderate amounts (3–4%) of acetic acid in silage had the advantage of suppressing yeasts during fermentation, hence enhancing silage stability when exposed to air (41). Here, the present study used FS to make silage that was well-fermented, and it had very low levels of propionic acid and butyric acid. Furthermore, clostridial organisms are responsible for the metabolism of soluble sugars or organic (lactic) acids to produce butyric acid, resulting in significant DM losses and inadequate energy recovery (40). Indeed, the current investigation demonstrated that conserving BC with FS inhibited the development of coliform bacteria, suggesting a greater effect for Clostridium in silages. The restricted development of Clostridium in silages might explain why certain silages in the current investigation exhibited undetectable amounts of propionic acid and butyric acid (41). Overall, FS has been proven to suppress undesirable organic pollutants and may be associated with sulfate-reducing bacteria such as Clostridium through oxidation processes involving ferrous ions (Fe^2+^) and hydrogen peroxide [H_2_O_2_; Soudham et al. (9)]. The current findings implied that the use of FS for ensiling lignocellulose agricultural biomass is critical because it has the potential to improve fermentative quality without significantly altering the chemical composition and to promote the development of beneficial ensiling bacteria by suppressing undesired ensiling bacteria. The most plausible explanation is that FS degrades those undesired ensiling microorganisms *via* cell wall or nucleic acid production. However, the mechanism by which these activities occur remains unknown, and further research is needed to understand how FS behaves in the presence of undesired ensiling microorganisms.

In general, a 21-day ensiling duration seems insufficient to achieve a steady silage quality. Practically, ensiling tropical grass can be carried out at least once a month to achieve silage quality stability (46). Additional *in vitro* research to acquire a better knowledge of the stability of iron sulfate with or without other natural or synthetic additives with a longer period of ensiling may provide more favorable results than this study.

### Anthocyanin in Black Cane Is Stable Throughout Rumen Fermentation

In general, using feedstuffs as a source of carbohydrate, protein, and fat, as well as oligomers and polymers of polyphenols (including anthocyanin), results in greater degradation of the compounds themselves during rumen incubation. Despite the contradictory findings in this study, our findings support Hosoda et al.'s conclusion that the unresponsive anthocyanin during rumen fermentation may be similar to anthocyanin-rich corn (43). That said, anthocyanin-rich corn is shielded from ruminal digestion as well and may therefore be absorbed by ruminant animals. Additional data from *in vivo* trials revealed that anthocyanin-rich purple corn stover silage improved the antioxidant status of goats as well as transferring anthocyanin composition to the milk and meat of the animals (4, 47). It is possible that the molecular weight of anthocyanin in feedstuffs has a functional effect on the performance of ruminant animals (4, 24). Similarly, our findings showed that incubating BC in fresh or ensiled form with ruminal fluid from small ruminants did not degrade the anthocyanin. It is clear that anthocyanin-rich corn, anthocyanin-rich purple corn stover silage, and BC in both its fresh and ensiled forms have different molecular weights. Our findings backed up the conclusions of the previous research (43). On the basis of these and other findings, the present study concluded that the anthocyanin in the BC is shielded from ruminal digestion and, as a consequence, might be absorbed by small ruminants. However, an *in vivo* trial employing small ruminants is essential to confirm the outcomes of these *in vitro* tests utilizing FS alone or in combination with additional additives.

### Ferrous Sulfate-Treated Silage Enhanced Total VFA Concentrations as Well as Contributes to Sustainable Mitigation of Ruminal Biogas Methane

The present study shown that after 24 h of ruminal fermentation, the concentrations of ammonia nitrogen and pH of incubated pre-ensiled materials and BC silages were consistent, but not the concentrations of total VFAs (Figure 3). FBC and SHF showed greater VFA concentrations after 24 h of ruminal fermentation than SZF and SLF. Despite the fact that both pre-ensiled materials and BC silages generated the same amount of propionate and butyrate, only FBC and SHF enhanced the amount of acetic acid in the rumen after 24 h of incubation when compared to SZF and SLF. This resulted in an increase in the acetic to propionic acid ratio, as with FBC and SHF (Figure 3). Our findings were consistent with prior studies (2, 7), indicating that FS has a minor residual influence and that BC may maintain an acceptable acid-base environment without inducing rumen acidosis (pH = 6.70–6.86). Greater substrate breakdown led to higher VFA concentrations in rumen fluids. Ruminant animals require VFAs as energy sources since they are essential organic acids for their survival. It is well known that flavonoid-rich biomass may change rumen VFAs in goat rumen fluids (25). The two most often synthesized VFAs are acetate and butyrate. This is due to the fact that the hydroxyl groups in anthocyanins are the principal functional groups metabolized in the rumen through glycoside hydrolysis and heterocyclic compound cleavages (3). As a result, our findings may be compatible with previous studies (2, 43), which demonstrated that anthocyanin-rich plants or biomass altered VFA fermentation by degrading substrate fermentation and accumulating largely as acetic acid. However, our data indicated that no change in fermentation occurred for propionic acid or butyric acid, which might account for the rise in total VFAs as a result of increased acetic acid causing a larger degree of soluble carbohydrates breakdown, particularly cellulose degradation. Consistent with our findings, Hosoda et al. (43) and Tian et al. (2) revealed that ruminal fluid acetic acid increased in response to anthocyanin-rich maize feeding in ruminant animals. This might be explained by anthocyanins' role in bacterial population regulation, as shown by shifting VFA production and other fermentation gases such as ruminal biogas methane and carbon dioxide, as well as an alternate hydrogen sink (2, 29, 30). According to a recent study, optimizing VFAs production might be accomplished by matching the needs of the rumen host to the needs of the rumen microbiome and the availability of fermentable substrate in rumen fluids (48). Overall, our findings, in conjunction with previously published data, showed that differences in VFA production were caused by anthocyanin sources, anthocyanin delivery, fermentation substrates, and animal physiological phases.

Rumen bacteria's anaerobic breakdown of cellulolytic and hemicellulolytic feedstuffs is intimately related to the presence of enteric ruminal gases such as hydrogen, methane, and carbon dioxide (48). In this study, Figure 3 shows that residual FS in BC had an influence on ruminal biogases. Incubation of ensiled forms (SZF) and ensiled forms with low FS ensiling (SLF) resulted in a higher drop in total gas production, whereas incubation of ensiled forms with high FS ensiling (SHF) resulted in a positive trend nearly identical to that of FBC, generating total gas for 24 h. Simultaneously, SHF and FBC did not produce significantly different amounts of carbon dioxide, suggesting that they produced similar patterns of total fermentable substrate in addition to carbon dioxide yields. This was not unexpected, considering that the carbohydrate and glucose levels of SHF and FBC were essentially identical throughout substrate fermentation (Table 1). Our results were comparable with those of a previous study (49) that investigated the addition to carbon dioxide yields of many tropical and temperate forages with high nonfibrous carbohydrate content and low lignin concentration.

Moreover, after a 24-h rumen fermentation, SHF produced less methane gas than FBC or SZF in this study (Figure 3). Our findings suggested that the residual effect of FS on BC silage may be due to rumen methanogenesis interference. Methanogenesis is the primary biochemical mechanism that converts the metabolic hydrogen generated during carbohydrate fermentation in the rumen into methane gas. One of the most effective strategies to limit methane emissions is employing a chemical substance and the appropriate amount of hydrogenotrophs that use the hydrogen-electron sink route, such as nitrate and sulfate (50, 51). SHF, as previously stated, generated higher total VFAs and acetic acid from more fermentable carbohydrates in the rumen fluids. Because of the residual impact of FS, existing rumen acetogen populations seemed to utilize more carbon dioxide and hydrogen during the hydrogen-using metabolic pathway. Methanogenesis in the rumen may have outcompeted reductive acetogenesis or the conversion of carbon dioxide and hydrogen to acetate, with acetogens having a functional advantage over methanogens in the rumen since acetogens survive predominantly by metabolizing carbohydrates (51).

Other research on employing anaerobic digestion to clean up sulfate-containing waste has been published. Iron seems to impede methanogenesis by making electron exchange more difficult for iron-reducing bacteria and methanogens containing Fe oxides (52). According to our findings, residual sulfate combined with plant polyphenols effectively limits methane generation *in vitro* by rumen cultures while not interfering with feed digestion, fermentation, or microbial populations (53–55). Surpassing the sulfate threshold (0.3–0.4% sulfur as sulfate) has been demonstrated to have a more detrimental influence on animal performance (55). Based on the findings presented, BC containing 0.030% FS (SHF) seems to have the capacity to suppress methane emissions, but additional *in vivo* testing is required.

### Ferrous Sulfate-Treated Silage Modulates Cellulolytic Bacteria, and Suppresses Methanogenic Bacteria in Rumen Fluid

The present study shown that raising the number of *R. albus*, lowering the number of methanogenic bacteria, and maintaining the proportion of total bacteria, *F. succinogenes, B. fibrisolvens, M. elsdenii, S. bovis*, and protozoa resulted in equivalent performance for both FBC and SHF at three time periods (6, 12, 24 h) during *in vitro* rumen incubation. Our findings corroborated a previous study (2), in which it was shown that fermented substrates rich in anthocyanin had a beneficial effect on cellulolytic activity in relation to modulating major fiber-degraders in the rumen harbor. The absence of anthocyanin BC impacts on *R. flavefaciens and F. succinogenes* might be attributed to differences in the substrate fermentation employed, anthocyanin molecular weight, feedstuff processing, and animal type. For example, Niu et al. (56) observed that the average abundances of *R. albus* and *R. flavefaciens* in the rumen fluid of finishing steers were largely similar, while the average abundance of *F. succinogenes* was significantly greater in ensiled mulberry leaves or sun-dried mulberry fruit pomace richer in flavonoids and anthocyanins compared with the normal mixed diet (no anthocyanins). However, Yusuf et al. (57) observed that *Andrographis paniculata* leaves rich in plant active substances (lactones, anthocyanin, flavonoids, sterols) in goats' diet had a propensity to enhance the number of ruminal *R. albus* and *R. flavefaciens* while leaving the ruminal fluid total bacteria unaltered, allowing goats to improve their nutrient digestibility. As a consequence, our results revealed that the availability of larger quantities of digestible carbohydrates and/or total soluble solids (particularly glucose, sucrose, and fructose) in FBC and SHF compared with those in SZF and SLF may have contributed to a higher number of *R. albus*, resulting in variations in VFA concentrations (shown above). However, most of the cellulolytic-degraders' effects were found only in *R. albus* throughout the 24 h of incubation, showing that anthocyanin could modify only *R. albus* during cellulolytic activity in the rumen harbor throughout the experiment. This was validated by multi-omic studies using gnotobiotic sheep where *R. albus, R. flavefaciens*, and *F. succinogenes* competed for cellulosic biomass (58).

By focusing on protozoa-associated methanogens, (55) reviewed a thorough knowledge base of ruminal methanogens and their responses to ruminal bacteria. The decreased amount of 16S *Methanobacterium* rRNA gene sequences identified from protozoa suggested that protozoa-associated methanogens are not linked to *Methanobacterium* (e.g., methanogenic bacteria). Essentially, plant flavonoid-anthocyanin has been found to have substantial antibacterial activity by interfering with the semipermeable barrier of methanogens during intercellular contacts; however, the efficiency of that plant flavonoid-anthocyanin is dependent on molecular weight, dosages, type, sources, and the basal substrate employed (2, 16, 25). Over the last several years, iron-reducing bacteria have been widely utilized to inhibit methane producers, particularly methanogens, during anaerobic digestion, and this method has become more effective (52). The possibility of direct electron transfer between iron-reducing bacteria and methanogens through iron oxides might explain why methanogens were eradicated. Therefore, our findings employing FS as residual iron in SLF and SHF may have an impairing impact on free-living ruminal methanogens and might be deemed anti-methanogenic characteristics.

## Conclusions

Black cane (BC, *Saccharum sinensis* Robx.) is a potential ruminant feedstock because of its high concentration of soluble carbohydrates and anthocyanins, along with high lignification or silicification of biomass. Increasing dilutions of ferrous sulfate heptahydrate (FS) added to silages resulted in a linear decrease in dry matter, anthocyanin, and nutritional value losses when ensiling BC for 21 d of aerobic exposure. The lignin values decreased linearly as the proportion of FS increased up to 0.030%. The addition of FS up to 0.030% resulted in an ideal balance of ensiling properties in ruminants, with no negative effect on rumen fermentation with lowering ruminal methane gases. The findings of this study can facilitate the creation of a strategy for developing ruminant diets based on roughage. This will be beneficial to the environment as well. Since the present study was performed over a short ensiling time, further *in vitro* research to gain a better understanding of the combination of iron sulfate with other natural or synthetic additives over a longer period of ensiling may yield better results; *in vivo* studies and feeding trials in ruminants and/or small ruminants are required to understand the mechanism by which anthocyanins alter digestibility, fermentation, antioxidant property, rumen microscopic and rumen-derived products in the absence or presence of residual FS.

## Data Availability Statement

The original contributions presented in the study are included in the article/Supplementary Material, further inquiries can be directed to the corresponding author/s.

## Ethics Statement

The animal study was reviewed and approved by the Animal Ethics Committee of Suranaree University of Technology issued a statement approving the experimental protocol (SUT 4/2558). The research was carried out in accordance with regulations on animal experimentation and the Guidelines for the Use of Animals in Research as recommended by the National Research Council of Thailand (U1-02632-2559). Written informed consent was obtained from the owners for the participation of their animals in this study.

## Author Contributions

NS: conceptualization, methodology, formal analysis, investigation, resources, data curation, writing—review and editing, visualization, and project administration. SP: conceptualization, methodology, writing—review and editing, supervision, project administration, and funding acquisition. AS: conceptualization, methodology, and writing—review and editing. PP: conceptualization, methodology, formal analysis, resources, data curation, writing—review and editing, supervision, project administration, and funding acquisition. RP: conceptualization, methodology, formal analysis, investigation, resources, data curation, writing—original draft preparation, writing—review and editing, visualization, supervision, project administration, and funding acquisition. All authors contributed to the article and approved the submitted version.

## Funding

This research was funded by Suranaree University of Technology (SUT; contract no. Full-time 61/02/2021), Thailand Science Research and Innovation (TSRI), National Science Research and Innovation Fund (NSRF; project codes: 90464; 160368; FF3-303-65-36-17), National Research Council of Thailand (NRCT; project code: 900105), and Nakhon Ratchasima Rajabhat University (NRRU).

## Conflict of Interest

The authors declare that the research was conducted in the absence of any commercial or financial relationships that could be construed as a potential conflict of interest.

## Publisher's Note

All claims expressed in this article are solely those of the authors and do not necessarily represent those of their affiliated organizations, or those of the publisher, the editors and the reviewers. Any product that may be evaluated in this article, or claim that may be made by its manufacturer, is not guaranteed or endorsed by the publisher.

## References

[1] Grivet L Glaszmann JC D'Hont A. 3. Molecular Evidence of Sugarcane Evolution and Domestication. In: Darwin's Harvest. Columbia University Press (2006). p. 49-66. 10.7312/motl13316-004

[2] Tian XZ Li JX Luo QY Zhou D Long QM Wang X. Effects of purple corn anthocyanin on blood biochemical indexes, ruminal fluid fermentation, and rumen microbiota in goats. Front Vet Sci. (2021) 8:715710. 10.3389/fvets.2021.715710 34589534 PMC8475905

[3] Purba RAP Paengkoum S Yuangklang C Paengkoum P Salem AZM Liang JB. Mammary gene expressions and oxidative indicators in ruminal fluid. Blood, milk, and mammary tissue of dairy goats fed a total mixed ration containing piper meal (*Piper betle* L). Ital J Anim Sci. (2022) 21:129–41. 10.1080/1828051X.2021.2007173

[4] Tian XZ Lu Q Zhao S Li J Luo Q Wang X. Purple corn anthocyanin affects lipid mechanism, flavor compound profiles, and related gene expression of *longissimus thoracis* et lumborum muscle in goats. Animals. (2021) 11:2407. 10.3390/ani11082407 34438864 PMC8388639

[5] Xu P Cheng S Han Y Zhao D Li H Wang Y. Natural variation of lignocellulosic components in miscanthus biomass in china. Front Chem. (2020) 8:1028. 10.3389/fchem.2020.595143 33251186 PMC7674668

[6] Vorlaphim T Paengkoum P Purba RAP Yuangklang C Paengkoum S Schonewille JT. Treatment of rice stubble with *Pleurotus ostreatus* and urea improves the growth performance in slow-growing goats. Animals. (2021) 11:1053. 10.3390/ani11041053 33917899 PMC8068234

[7] Suong NTM Paengkoum S Schonewille JT Purba RAP Paengkoum P. Growth performance, blood biochemical indexes, rumen bacterial community, and carcass characteristics of goats fed anthocyanin-rich black cane silage. Front Vet Sci. (2022) 9:880838. Available online at: https://www.frontiersin.org/articles/10.3389/fvets.2022.880838 35573401 10.3389/fvets.2022.880838 PMC9101464

[8] Zhao J Zhang H Zheng R Lin Z Huang H. The enhancement of pretreatment and enzymatic hydrolysis of corn stover by FeSO[[sb]]4[[/s]] pretreatment. Biochem Eng J. (2011) 56:158–64. 10.1016/j.bej.2011.06.002

[9] Soudham VP Brandberg T Mikkola JP Larsson C. Detoxification of acid pretreated spruce hydrolysates with ferrous sulfate and hydrogen peroxide improves enzymatic hydrolysis and fermentation. Bioresour Technol. (2014) 166:559–65. 10.1016/j.biortech.2014.05.096 24953967

[10] Bao H Sagues WJ Wang Y Peng W Zhang L Yang S. Depolymerization of lignin into monophenolics by ferrous/persulfate reagent under mild conditions. ChemSusChem. (2020) 13:6582–93. 10.1002/cssc.202002240 33078554

[11] Wang Y Jiang M Zhang Z Sun H. Effects of over-load iron on nutrient digestibility, haemato-biochemistry, rumen fermentation and bacterial communities in sheep. J Anim Physiol Anim Nutr. (2020) 104:32–43. 10.1111/jpn.13225 31663652

[12] FEEDAP. Safety and efficacy of iron compounds (E1) as feed additives for all animal species: ferrous carbonate; ferric chloride, hexahydrate; ferrous fumarate; ferrous sulphate, heptahydrate; ferrous sulphate, monohydrate; ferrous chelate of amino acids, hydrate; ferrous chelate of glycine, hydrate, based on a dossier submitted by FEFANA asbl. EFSA J. (2016) 14:4396. 10.2903/j.efsa.2016.4396 PMC13093174 42016893

[13] AOAC. Official Methods of Analysis. Gaitherburg, Maryland. USA: AOAC International Suite (2005). p. 500.

[14] Van Soest PJ Robertson JB Lewis BA. Methods for dietary fiber, neutral detergen fiber, and nonstarch polysaccharides in relation to animal nutrition. J Dairy Sci. (1991) 74:3583–97. 10.3168/jds.S0022-0302(91)78551-2 1660498

[15] Cai Y Du Z Yamasaki S Nguluve D Tinga B Macome F. Community of natural lactic acid bacteria and silage fermentation of corn stover and sugarcane tops in Africa. Asian-Australas J Anim Sci. (2020) 33:1252–64. 10.5713/ajas.19.0348 32054211 PMC7322639

[16] Li X Yao S Tu B Li X Jia C Song H. Determination and comparison of flavonoids and anthocyanins in Chinese sugarcane tips, stems, roots and leaves. J Sep Sci. (2010) 33:1216–23. 10.1002/jssc.200900567 20235128

[17] Tian XZ Paengkoum P Paengkoum S Thongpea S Ban C. Comparison of forage yield. Silage fermentative quality, anthocyanin stability, antioxidant activity, and in vitro rumen fermentation of anthocyanin-rich purple corn (Zea mays L) stover and sticky corn stover. J Integr Agric. (2018) 17:2082–95. 10.1016/S2095-3119(18)61970-7

[18] Purba RAP Paengkoum P. Bioanalytical HPLC method of Piper betle L. for quantifying phenolic compound, water-soluble vitamin, and essential oil in five different solvent extracts. J Appl Pharm Sci. (2019) 9:033–9. 10.7324/JAPS.2019.90504

[19] Song TH Han OK Park T Kim DW Yoon C Kim KJ. Anthocyanin stability and silage fermentation quality of colored barley. J Kor Grassl Forage Sci. (2012) 32:335–42. 10.5333/KGFS.2012.32.4.335

[20] Purba RAP Paengkoum S Paengkoum P. Development of a simple high-performance liquid chromatography-based method to quantify synergistic compounds and their composition in dried leaf extracts of *Piper sarmentosum* Robx. Separations. (2021) 8:152. 10.3390/separations8090152

[21] Fawcett JK Scott JE. A rapid and precise method for the determination of urea. J Clin Pathol. (1960) 13:156. 10.1136/jcp.13.2.156 13821779 PMC480024

[22] Paengkoum S Tatsapong P Taethaisong N Sorasak T Purba RAP Paengkoum P. Empirical evaluation and prediction of protein requirements for maintenance and growth of 18–24 months old thai swamp buffaloes. Animals. (2021) 11:1405. 10.3390/ani11051405 34069134 PMC8156132

[23] Kozaki M Uchimura T Okada S. Experimental manual of lactic acid bacteria. Asakurasyoten, Tokyo, Japan. (1992). p. 34–7.

[24] Paengkoum S Petlum A Purba RAP Paengkoum P. Protein-binding affinity of various condensed tannin molecular weights from tropical leaf peel. J Appl Pharm Sci. (2021) 11:114–20. 10.7324/JAPS.2021.110314

[25] Purba RAP Yuangklang C Paengkoum S Paengkoum P. Milk fatty acid composition, rumen microbial population and animal performance in response to diets rich in linoleic acid supplemented with Piper betle leaves in Saanen goats. Anim Prod Sci. (In Press). 10.1071/AN20182

[26] Menke KH Steingass H. Estimation of the energetic feed value obtained from chemical analysis and *in vitro* gas production using rumen fluid *Anim*. Res Dev. (1988) 28:7–55.

[27] Orskov ER Mcdonald I. The estimation of protein degradability in the rumen from incubation measurements weighted according to rate of passage. J Agric Sci, Camb. (1970) 92:499–503. 10.1017/S0021859600063048

[28] Purba RAP Paengkoum S Yuangklang C Paengkoum P. Flavonoids and their aromatic derivatives in *Piper betle* powder promote *in vitro* methane mitigation in a variety of diets. Cienc Agrotec. (2020) 44:e012420. 10.1590/1413-7054202044012420

[29] Purba RAP Yuangklang C Paengkoum P. Enhanced conjugated linoleic acid and biogas production after ruminal fermentation with *Piper betle* L. supplementation Ciênc Rural. (2020) 50:e20191001. 10.1590/0103-8478cr20191001

[30] Purba RAP Yuangklang C Paengkoum S Paengkoum P. Piper oil decreases *in vitro* methane production with shifting ruminal fermentation in a variety of diets. Int J Agric Biol. (2021) 25:231–40. 10.17957/IJAB/15.1661

[31] Yu Z Morrison M. Improved extraction of PCR-quality community DNA from digesta and fecal samples. Biotechniques. (2004) 36:808–12. 10.2144/04365ST04 15152600

[32] Atikah IN Alimon AR Yaakub H Abdullah N Jahromi MF Ivan M. Profiling of rumen fermentation, microbial population and digestibility in goats fed with dietary oils containing different fatty acids. BMC Vet Res. (2018) 14:344. 10.1186/s12917-018-1672-0 30558590 PMC6297943

[33] Koike S Kobayashi Y. Development and use of competitive PCR assays for the rumen cellulolytic bacteria: *Fibrobacter succinogenes, Ruminococcus albus* and *Ruminococcus flavefaciens*. FEMS Microbiol Lett. (2001) 204:361–6. 10.1111/j.1574-6968.2001.tb10911.x 11731149

[34] Denman SE McSweeney CS. Development of a real-time PCR assay for monitoring anaerobic fungal and cellulolytic bacterial populations within the rumen. FEMS Microbiol Ecol. (2006) 58:572–82. 10.1111/j.1574-6941.2006.00190.x 17117998

[35] Zhang CM Guo YQ Yuan ZP Wu YM Wang JK Liu JX. Effect of octadeca carbon fatty acids on microbial fermentation, methanogenesis and microbial flora *in vitro*. Anim Feed Sci Tech. (2008) 146:259–69. 10.1016/j.anifeedsci.2008.01.005

[36] Oba M. Review: Effects of feeding sugars on productivity of lactating dairy cows. Can J Anim Sci. (2011) 91:37–46. 10.4141/CJAS10069

[37] Li X Ma Z Yao S. Bioactivity-guided systematic extraction and purification supported by multitechniques for sugarcane flavonoids and anthocyanins. Food Bioprod Process. (2015) 94:547–54. 10.1016/j.fbp.2014.08.001

[38] Fernández AM Cabezuelo ABS de la Roza Delgado MB Arrojo MAG Gutiérrez AA. Modelling a quantitative ensilability index adapted to forages from wet temperate areas. Span J Agri Res. (2013) 11:455–62. 10.5424/sjar/2013112-3219

[39] Fly AD Czarnecki-Maulden GL Fahey GC Jr Titgemeyer EC. Hemicellulose does not affect iron bioavailability in chicks. J Nutr. (1996) 126:308–16. 10.1093/jn/126.1.308 8558316

[40] Pahlow G Muck RE Driehuis F Elferink SJWHO Spoelstra SF. Microbiology of ensiling. in Silage Science and Technology. (2003). p. 31–93. 10.2134/agronmonogr42.c2

[41] Kung L Shaver RD Grant RJ Schmidt RJ. Silage review: Interpretation of chemical, microbial, and organoleptic components of silages. J Dairy Sci. (2018) 101:4020–33. 10.3168/jds.2017-13909 29685275

[42] Mordenti AL Giaretta E Campidonico L Parazza P Formigoni A. A review regarding the use of molasses in animal nutrition. Animals. (2021) 11. 10.3390/ani11010115 33430515 PMC7827330

[43] Hosoda K Eruden B Matsuyama H Shioya S. Silage fermentative quality and characteristics of anthocyanin stability in anthocyanin-rich corn (*Zea mays L*.). Asian-Australas J Anim Sci. (2009) 22:528–33. 10.5713/ajas.2009.80525

[44] Pace E Jiang Y Clemens A Crossman T Rupasinghe HPV. Impact of thermal degradation of cyanidin-3-o-glucoside of haskap berry on cytotoxicity of hepatocellular carcinoma hepg2 and breast cancer mda-mb-231 cells. Antioxidants. (2018) 7:24. 10.3390/antiox7020024 29382057 PMC5836014

[45] Li W Gu M Gong P Wang J Hu Y Hu Y. Glycosides changed the stability and antioxidant activity of pelargonidin. LWT. (2021) 147:111581. 10.1016/j.lwt.2021.111581

[46] Kaewpila C Khota W Gunun P Kesorn P Cherdthong A. Strategic addition of different additives to improve silage fermentation, aerobic stability and *in vitro* digestibility of napier grasses at late maturity stage. Agriculture. (2020) 10:262. 10.3390/agriculture10070262

[47] Tian XZ Paengkoum P Paengkoum S Chumpawadee S Ban C Thongpea S. Purple corn (*Zea mays* L.) stover silage with abundant anthocyanins transferring anthocyanin composition to the milk and increasing antioxidant status of lactating dairy goats. J Dairy Sci. (2019) 102:413–8. 10.3168/jds.2018-15423 30415857

[48] Newbold CJ Ramos-Morales E. Review: ruminal microbiome and microbial metabolome: effects of diet and ruminant host. Animal. (2020) 14:s78–86. 10.1017/S1751731119003252 32024572

[49] Oliveira JGD Henrique DS Abreu MLC Fluck AC Mayer LRR Costa OAD. Evaluation of mathematical models to describe gas production kinetics of some tropical and temperate forages. Rev Bras Zootec. (2020) 49. 10.37496/rbz4920190197

[50] Greening C Geier R Wang CJ Woods LC Morales SE McDonald MJ. Diverse hydrogen production and consumption pathways influence methane production in ruminants. ISME J. (2019) 13:2617–32. 10.1038/s41396-019-0464-2 31243332 PMC6776011

[51] Ungerfeld EM. Metabolic hydrogen flows in rumen fermentation: Principles and possibilities of interventions. Front Microbiol. (2020) 11:589. 10.3389/fmicb.2020.00589 32351469 PMC7174568

[52] Jin Z Zhao Z Zhang Y. Insight into ferrihydrite effects on methanogenesis in UASB reactors treating high sulfate wastewater: reactor performance and microbial community. Environ Sci Water Res Technol. (2020) 6:1794–803. 10.1039/D0EW00154F

[53] Patra AK Yu Z. Combinations of nitrate, saponin, and sulfate additively reduce methane production by rumen cultures in vitro while not adversely affecting feed digestion, fermentation or microbial communities. Bioresour Technol. (2014) 155:129–35. 10.1016/j.biortech.2013.12.099 24440491

[54] Patra AK Yu Z. Effects of adaptation of *in vitro* rumen culture to garlic oil, nitrate, and saponin and their combinations on methanogenesis, fermentation, and abundances and diversity of microbial populations. Front Microbiol. (2015) 6. 10.3389/fmicb.2015.01434 26733975 PMC4686681

[55] Patra AK Park T Kim M Yu Z. Rumen methanogens and mitigation of methane emission by anti-methanogenic compounds and substances. J Anim Sci Biotechnol. (2017) 8:13–13. 10.1186/s40104-017-0145-9 28149512 PMC5270371

[56] Niu Y Meng Q Li S Ren L Zhou B Schonewille T. Effects of diets supplemented with ensiled mulberry leaves and sun-dried mulberry fruit pomace on the ruminal bacterial and archaeal community composition of finishing steers. PLoS ONE. (2016) 11:e0156836. 10.1371/journal.pone.0156836 27258373 PMC4892645

[57] Yusuf AL Adeyemi KD Samsudin AA Goh YM Alimon AR Sazili AQ. Effects of dietary supplementation of leaves and whole plant of Andrographis paniculata on rumen fermentation, fatty acid composition and microbiota in goats. BMC Vet Res. (2017) 13:349. 10.1186/s12917-017-1223-0 29178910 PMC5701315

[58] Yeoman CJ Fields CJ Lepercq P Ruiz P Forano E White BA. *In vivo* competitions between *Fibrobacter succinogenes, Ruminococcus flavefaciens*, and *Ruminoccus albus* in a gnotobiotic sheep model revealed by multi-omic analyses. MBio. (2021) 12:e03533–03520. 10.1128/mBio.03533-20 33658330 PMC8092306

