# Supplementary material for: Silage Fermentation Quality, Anthocyanin Stability, and in vitro Rumen Fermentation Characteristic of Ferrous Sulfate Heptahydrate-Treated Black Cane (Saccharum sinensis R.)
Source: Front Vet Sci. 2022 May 17;9:896270. doi: 10.3389/fvets.2022.896270 (PMC9152447; doi:10.3389/fvets.2022.896270)
Supplement: Supplementary file 1 [file Table_1.DOCX]

Supplementary Material

**TABLE S1 Primers used in the present study**

| Items | Primer sequence (5'- 3') | Temperature (°C) |
| --- | --- | --- |
| Total bacteria | F: CGGCAACGAGCGCAACCC | 55 |
|  | R: CCATTGTAGCACGTGTGTAGCC |  |
| *Ruminococcus albus* | F: CCCTAA AAGCAG TCTTAGTTCG | 55 |
|  | R: CCTCCTTGCGGTTAGAACA |  |
| *Ruminococcus flavefaciens* | F: TCTGGAAACGGATGGTA | 60 |
|  | R: CCTTTAAGACAGGAGTTTACAA |  |
| *Fibrobacter succinogens* | F: GTTCGGAATTACTGGGCGTAAA | 55 |
|  | R: CGCCTGCCCCTGAACTATC |  |
| *Butyrivibrio fibrisolvens* | F: ACACACCGCCCGTCACA | 58 |
|  | R: TCCTTACGGTTGGGTCACAGA |  |
| *Megasphaera elsdenii* | F: GACCGAAACTGCGATGCTAGA | 59 |
|  | R: CGCCTCAGCGTCAGTTGTC |  |
| *Streptococus bovis* | F: GCCA GGCTATTTAGGTGACACTATAG | 58 |
|  | R: GGGT AATACGACTCACTATAGGG |  |
| Methanogen | F: TTCGGTGGATCDCARAGRGC | 58 |
|  | R: GBARGTCGWAWCCGTAGAATC |  |
| Protozoa | F: CTTGCCCCTCYAATCGTWCT | 55 |
|  | R: GCTTTCGWTGGTAGTGTATT |  |
